# Improved healing response in delayed unions of the tibia with low-intensity pulsed ultrasound: results of a randomized sham-controlled trial

**DOI:** 10.1186/1471-2474-11-229

**Published:** 2010-10-08

**Authors:** Markus D Schofer, Jon E Block, Julia Aigner, Andreas Schmelz

**Affiliations:** 1 Department of Orthopaedics, University Hospital Marburg, Baldingerstrasse, 35043 Marburg, Germany; 2 2210 Jackson Street, Suite 401, San Francisco, CA 94115, USA; 3 Department of Trauma, Hand, and Reconstructive Surgery, University of Ulm, Steinhövelstrasse 9, 89075 Ulm, Germany

## Abstract

**Background:**

We compared the healing response of tibial delayed unions between subjects treated with low-intensity pulsed ultrasound (LIPUS) (n = 51) and subjects treated with a sham device (n = 50). Fracture age was ≥ 4 months in all cases. Study personnel and participants were blinded to random treatment assignment throughout the study.

**Methods:**

This multi-center randomized sham-controlled trial was undertaken at six hospitals in Germany. Adult patients who had sustained a tibial shaft fracture that subsequently showed inadequate progress toward healing (i.e., delayed union) were enrolled and randomized to receive either LIPUS (Exogen 2000/2000+, Smith & Nephew GmbH, Schenefeld, Germany) or an identical nonoperative sham device. The daily treatment duration was 20 minutes, for a period of 16 weeks. Subjects randomly assigned to active treatment had the ultrasound pressure wave signal set at the following parameters: 1.5 MHz frequency, 1 kHz repetition rate, 200 μs pulse duration, 30 mW/cm^2 ^spatial intensity. Progress toward healing was estimated from changes in bone mineral density (BMD) and gap area as determined from computed tomography scans. Intention-to-treat analysis was conducted using a multiple imputation methodology.

**Results:**

Based on log-transformed data, mean improvement in BMD was 1.34 (90% confidence interval (CI) 1.14 to 1.57) times greater for LIPUS-treated subjects compared to sham (p = 0.002). A mean reduction in bone gap area also favored LIPUS treatment (p = 0.014).

**Conclusions:**

These findings demonstrate significantly greater progress toward bone healing after LIPUS treatment compared to no LIPUS treatment in subjects with established delayed unions of the tibia.

## Background

Low-intensity pulsed ultrasound (LIPUS) is a safe, non-invasive treatment option that has been proven to enhance the healing of fresh closed tibial fractures in a Level-I randomized sham-controlled trial [1]. These results have been corroborated in studies of complex open fractures, prone to delayed union or nonunion [2,3]. However, until recently, the specific mechanism of action of LIPUS on the bone healing process remained uncertain and a matter of intense speculation, discussion and debate [4-6]. Using quantitative histomorphometric analysis of biopsy samples from fibular delayed unions, Rutten et al [7] reported that use of LIPUS accelerates fracture healing by directly increasing bone formation through increased osteoblastic activity. These authors showed a demonstrable increase, compared to sham-treated control patients, in osteoid thickness, mineral apposition rate and bone volume at the leading edge of new bony callus formation.

A number of positive single arm studies of LIPUS provides additional clinical evidence that the accelerated healing achieved in fresh fractures can be extended to the management of delayed unions and nonunions of long bones [8-12]. However, due to ethical considerations and difficulty in recruiting subjects, randomized sham-controlled trials in this setting to definitively demonstrate the value of LIPUS have been absent [6].

The current study is the first randomized sham-controlled trial of LIPUS in the treatment of delayed unions of the tibia, offering Level-I evidence of effectiveness. We tested the hypothesis that in comparison to a placebo, 16 consecutive weeks of LIPUS treatment would accelerate the progression to healing as evidenced by quantitative radiographic measurements of bone mineral density (BMD) and the reduction in the size of the residual gap area.

## Methods

### Patients

A multi-center, randomized, controlled trial was undertaken at six hospitals in Germany to determine the effectiveness of LIPUS in accelerating the healing process in delayed unions of the tibial shaft. All adult patients who had sustained a tibial shaft fracture that subsequently showed inadequate progress toward healing (i.e., delayed union) and provided informed consent were candidates for inclusion in the study. Delayed union was defined as lack of clinical and radiologic evidence of union, bony continuity or bone reaction at the fracture site for no less than 16 weeks from the index injury or the most recent intervention [6,9,13]. Standard anteroposterior (AP) and lateral radiographs were performed at baseline to establish lack of union. Patients were not admitted to the study if any of the following criteria were present: pregnancy, revision or reoperations at the fracture site within 16 weeks of enrollment, deep wound infection, excessive malalignment. The trial was approved by the institutional review boards at each clinical site and was conducted in adherence to the Declaration of Helsinki.

One hundred one subjects (age range: 14 to 70 years) with delayed unions of the tibia, enrolled between January 2002 and December 2005, were randomly allocated to treatment with either an active LIPUS device (n = 51) or an inactive sham device (n = 50).

### Interventions

Patients with established delayed unions of the tibia were randomized to receive either LIPUS or an identical nonoperative sham device. The study device was the Exogen 2000/2000+ (Smith & Nephew GmbH, Schenefeld, Germany). The sham device was inactivated in such a manner as to be indistinguishable from the active LIPUS device, except that it did not emit acoustic pressure waves.

All subjects were instructed to use the device for 20 minutes per day, for 16 weeks. Those subjects randomly assigned to active treatment had the ultrasound pressure wave signal set at the following parameters: 1.5 MHz frequency, 1 kHz repetition rate, 200 μs pulse duration, 30 mW/cm^2 ^spatial intensity. All devices recorded the amount of time per day of use as a means of assessing subject compliance with the treatment protocol.

Treatment was assigned randomly to each subject on a 1:1 basis in blocks of six and randomization was stratified within each clinical site. The randomization code was developed using a computer random number generator. The investigators, subjects and sponsor were blinded to the random allocation sequence prior to initiation of treatment and throughout the entire duration of this study. Once the study was complete and the last subject reached 16 weeks of follow-up, the randomization code was broken and treatment assignments revealed to the study statistician. Quantitative radiographic assessments of BMD and gap area also were undertaken without knowledge of treatment group assignment.

### Outcomes

The primary outcomes of this study were BMD and gap area at the fracture site, assessed by computed tomography (CT) scanning. All radiographic imaging studies were evaluated at a central Radiology laboratory using a dedicated workstation for image processing and digitization. BMD was determined for three regions of interest: fracture site, 2-3 mm proximal and distal. BMD also was determined in a healthy reference area. For each of the three regions, three replicate observations were obtained. These three replicates were averaged to obtain a mean BMD for each region separately for pre-treatment and post-treatment evaluations. Region specific changes from baseline were then computed. Finally, the three region specific changes from baseline obtained for each subject were averaged to obtain the primary effectiveness endpoint defined as the mean change from baseline in BMD.

BMD was indirectly estimated using the mean CT attenuation coefficients, or Hounsfield units (HUs), for each region of interest. Calibration to varying concentrations of K_2_HPO_4 _to directly quantitate BMD in equivalent mineral density (mg/cm^3^) was not undertaken. Thus, BMD results are reported in HUs [14,15]. Gap area in mm^2 ^was estimated directly from CT scans using digitized images.

The primary endpoint with respect to efficacy was change in BMD between pre-treatment and 16 weeks. Change in gap area at the fracture site was a secondary endpoint.

AP and lateral radiographs also were taken at 1, 2 and 3 month follow-up intervals. Blinded to treatment assignment, participating physicians were asked to judge the healing status (healed/not healed) of each study subject at 16 weeks.

### Statistical Methods

All analyses employed Statistical Analysis Software (SAS). Subject baseline characteristics were summarized using frequency and percentage distributions or descriptive statistics, as appropriate. Proportions were compared using the Chi-square test with Yates' continuity correction or Fisher's exact test. Continuous variables were compared using the two sample t-test.

The primary analysis was intention-to-treat (ITT) and involved all subjects who received random treatment assignments and initiated device usage. Seventeen subjects had missing post-treatment outcomes, consequently 84 subjects were included in descriptive analyses of 'completers'. There was notable differential drop-out between groups with 24% (12 of 50) of sham-treated subjects and 9.8% (5 of 51) of active-treated subjects missing post-treatment BMD values. The ITT cohort was preserved by imputing missing clinical endpoints using a multiple imputation procedure that minimizes bias from differential drop-outs and properly accounts for uncertainty in imputed values when performing statistical inference. Multiple imputation uses multiple predictions of missing clinical endpoints based on patient characteristics and the baseline value of the clinical outcome variables [16]. For each of five stochastically completed data sets, analysis of covariance (ANCOVA) was used to estimate a treatment group contrast that controlled for the baseline value of the clinical endpoint as well as clinical site. This ANCOVA approach is consistent with ICH E9 guidelines for statistical analysis of a multi-site clinical trial [17]. The estimated group difference obtained for each of the five stochastically completed data sets were appropriately combined to obtain a single estimate of the intervention effect. Attention was paid to the validity of statistical model assumptions using graphical and other approaches. We found that BMD could be validly analyzed with or without log transformation, but that valid inference required log transformation for gap area. Analyses on log transformed data expresses group differences in terms of relative improvement which has a convenient clinical interpretation. Estimates of relative and absolute group differences were presented along with confidence intervals that accounted for the multiple imputation.

All primary effectiveness results using statistical modeling were based on a one-sided alpha = 0.05 with 90% confidence intervals (CIs). Preliminary descriptive analyses without imputations and descriptive assessments of effect size (ES) were two-sided with 95% CIs.

As specified *a priori*, study hypotheses were ordered such that the hypothesis concerning change in BMD was tested first. Only if the active device was shown to be superior to sham based on a one-sided test at α = 0.05 would the second test regarding changes in bone gap area be performed. This *a priori *ordering eliminated the need to adjust significance levels for multiple testing.

The success rate for physician judgment of fracture healing was compared between groups using Fisher's exact test. The statistical content of the manuscript was approved by an expert in medical statistics and epidemiology.

## Results

Inspection of background characteristics between study groups showed generally good balance achieved through randomization (Table 1). Sham control subjects had a greater average body mass index (p = 0.03). Additionally, there was a larger percentage of LIPUS-treated subjects with time since fracture ≥ 48 weeks (41% vs. 24%) and a smaller percentage with < 24 weeks (14% vs. 30%), although this comparison did not achieve statistical significance (p = 0.08). Overall, compliance with the treatment regimen was excellent, with a median total time of device usage of 2040 minutes out of a possible 2240 minutes (91%).

**Table 1:** Background Characteristics by Study Group

**Characteristic**	**LIPUS**	**Control**	
	**(n = 51)**	**(n = 50)**	**P Value**
			
**Age**, *mean ± SD, y*	42.6 ± 14.6	45.1 ± 11.9	0.35
			
**Female**, *n (%)*	**15 **(29)	**9 **(18)	0.24
			
**Body Mass Index**, *mean ± SD, kg/m^2^*	25.1 ± 4.0	27.1 ± 5.0	0.03
			
**Fracture Age**, *mean ± SD, weeks*	60.3 ± 61.0	46.4 ± 41.7	0.18
**Distribution of Fracture Age**, *n (%)*			
< 24 weeks 24 to < 36 weeks 36 to < 48 weeks ≥ 48 weeks	**7 **(14) **16 **(32) **7 **(14) **21 **(41)	**15 **(30) **12 **(24) **11 **(22) **12 **(24)	0.08
			
Mechanism of Injury*, *n (%)*			
High-energy trauma Low-energy trauma	**26 **(52) **24 **(47)	**26 **(52) **24 **(28)	1.00
			
**Open Fracture**, *n (%)*	**16 **(31)	**22 **(44)	0.40
**Surgical Treatment^†^**, *n (%)*			
Intramedullary nail^‡^ Locking screws External fixation Osteosynthesis plate Supplemental bone graph	**29 **(58) **20 **(39) **16 **(31) **19 **(37) **17 **(33)	**24 **(49) **14 **(28) **18 **(36) **19 **(38) **13 **(26)	0.42 0.29 0.68 1.00 0.52
			
**Smoking Status**, *n (%)*			
Non smoker Ex-smoker Current smoker	**20 **(39) **12 **(24) **19 **(37)	**15 **(30) **12 **(24) **23 **(46)	0.57

* One missing value LIPUS.

^† ^One missing value each, LIPUS and Control.

^‡ ^Includes primary and any follow-up procedures.

**Figure 1:**
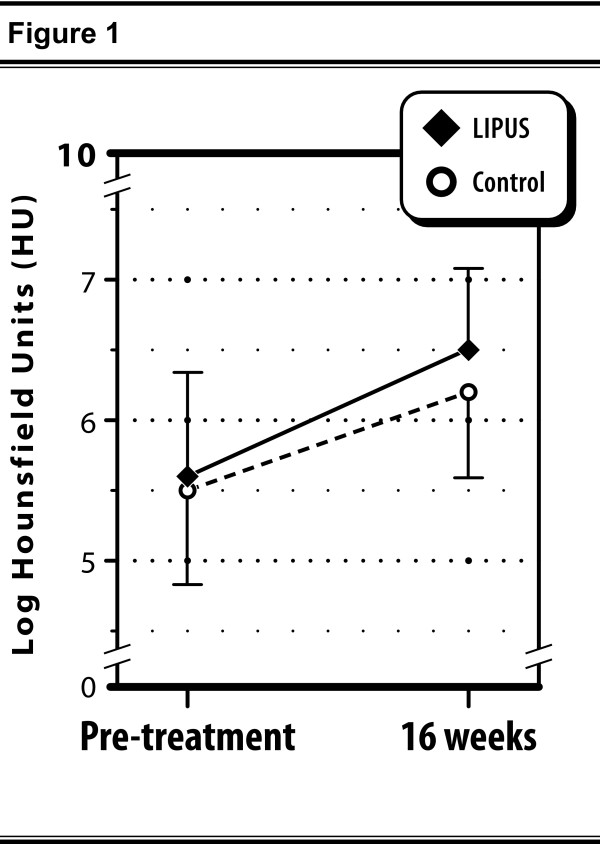
**Line graph illustrating improvement in bone mineral density for each treatment group separately through 16 weeks of follow-up**. Data based on 'completers' analysis of observed cases. The difference in mean improvements in log Hounsfield units was statistically significant (p = 0.014) with a corresponding effect size, 0.53, representing a medium degree of effectiveness.

Results from the descriptive 'completers' analysis of observed cases are expressed on the log scale in order to allow comparison of ES between BMD and gap area. The mean (SD) changes from pre-treatment to 16 weeks follow-up in log BMD were 0.87 (0.67) HU and 0.57 (0.38) HU for active- and sham-treated groups, respectively (t-test, p = 0.014) (Figure 1). The difference in these means, divided by the pooled standard deviation results in a standardized ES of 0.53 (95% CI 0.09 to 0.97). The corresponding mean changes (SD) in log gap area were -0.131 (0.072) mm^2 ^and -0.097 (0.070) mm^2 ^for active and sham groups, respectively (p = 0.034) resulting in a standardized effect size of comparable absolute value (ES = -0.47, 95% CI -0.91 to -0.03).

Following multiple imputation, the adjusted difference between active and sham study groups in the mean change in BMD was 122.4 HUs (90% CI 42.1 to 202.7). The null hypothesis of equal group mean changes in BMD was rejected with p = 0.007 (1-sided ANCOVA after multiple imputation).

Based on log transformed data, the adjusted mean improvement in BMD was 1.34 (90% CI 1.14 to 1.57) times greater for LIPUS-treated subjects compared to sham controls (p = 0.002).

A statistically significant benefit of LIPUS treatment also was realized in terms of mean reduction in bone gap area based on log transformed data using multiple imputation methods (1-sided, p = 0.014). The exponentiated difference in log mean changes was 0.974 (90% CI 0.956 to 0.993) reflecting proportionally smaller average gap area. For untransformed data, the group difference in mean adjusted changes from baseline in bone gap area was -0.457 mm^2 ^(90% CI -0.864 to -0.049) with 1-sided p = 0.03 similarly reflecting a smaller expected gap area in LIPUS-treated subjects compared to controls.

Post-treatment log BMD group differences were evaluated while controlling for baseline and other independent predictors using multiple regression analysis. Results demonstrated that the relative effectiveness of LIPUS remained statistically significant (p = 0.004) with negligible change in magnitude. After exponentiating the relevant parameter estimates, the adjusted mean BMD was found to be 1.35 (95% CI 1.10 to 1.65) times larger among patients treated with LIPUS compared to those treated with sham, which is nearly identical to the value obtained without covariate adjustment. Other independent variables entering this model at a statistically significant level included pre-treatment log BMD (ratio = 0.64, 95% CI 0.55 to 0.74, p < 0.0001), time since fracture < 48 weeks (ratio = 1.33, 95% CI 1.08 to 1.65, p = 0.009), and use of intramedullary nail fixation (ratio = 1.23, 95% CI 1.00 to 1.5, p = 0.047). For example, controlling for LIPUS status and these other variables, the (geometric) mean BMD was found to be 1.33 times larger for patients with a time since fracture < 48 weeks compared to 48 weeks or greater. Similarly, use of intramedullary nail fixation increases the predicted week 16 mean BMD by a factor of 1.23 (95% CI 1.00 to 1.50). Overall, the model accounted for approximately 41% of the variability in post-treatment log BMD. A time since injury by study group interaction added to this model was not significant (p = 0.76) suggesting that the bone growth benefit of LIPUS was present whether or not the time since fracture was 48 weeks or greater, despite the independent effects of time since fracture.

At the completion of the 16 week study period, 65% (33 of 51) of LIPUS and 46% (23 of 50) of sham subjects were judged to be healed by the participating physicians (p = 0.07). There were no device-related adverse events in this study group.

## Discussion

The prevalence of delayed union following tibial fracture has been estimated to be 4.4% [13]. Delayed unions, which often evolve into nonunions, can result in significant morbidity, functional impairment and loss of quality of life for the afflicted patient. Additional surgical interventions with supplemental bone grafting or use of bone growth factors are routinely required to assure healing once a nonunion is evident. These procedures are complex and costly [18]. There is consensus based on several systematic reviews and meta-analyses that use of LIPUS accelerates the healing of fresh fractures [5,19-21], and offers a cost-effective addition to conservative or operative management of these injuries [22].

The results of the current randomized controlled trial extend the positive findings of LIPUS treatment in fresh fractures and establish the effectiveness of this non-invasive modality in delayed unions of the tibia. This is the first study to offer Level-I evidence of this effect in a single fracture type. The primary conclusion from our efficacy analysis using multiple imputation was that the estimated increase in BMD among subjects randomized to active LIPUS treatment was 34% larger than among subjects randomized to receive sham treatment. The computed effect size based on the 'completers' cohort was 0.53 (95% CI 0.09 to 0.97) representing a medium degree of effectiveness [23]. Use of LIPUS also resulted in a significantly smaller residual gap area at the fracture site compared to sham treatment with comparable absolute magnitude of effectiveness (ES = -0.47, 95% CI -0.91 to -0.03). Multiple imputation was used to construct primary ITT assessments of relative effectiveness that allowed inclusion of all randomized subjects with comparable results. From these analyses it was estimated that the expected BMD at week 16 to be 1.34 (90% CI 1.14 to 1.57) times larger among patients treated with LIPUS compared to those treated with sham. Nearly identical results were obtained after controlling for other significant baseline covariates.

These findings have important implications for the management of tibial delayed unions and nonunions as these injuries account for 35% to 65% of all nonunions [24]. Also noteworthy, was the finding that long times since fracture (e.g., ≥ 48 weeks) were associated with poorer radiographic outcomes independent of treatment group. This relationship has been shown previously [9] and underscores the need to initiate LIPUS treatment at the earliest interval when delayed union is suspected.

The institutional review boards at the participating clinical centers limited the study to 16 weeks for ethical reasons. It is unlikely that most established delayed unions will heal completely in this time frame or show discernible improvement in patient reported outcomes [13]. Therefore, we measured progression to healing using surrogate measures of healing, BMD and gap area, because efficacy with LIPUS had previously been established in a Level-I randomized controlled trial of fresh long bone fractures [1] as well as in delayed unions and nonunions reported in a large patient registry [5,10], and in several single arm studies [8-12]. Indeed, quantitative CT measurements of BMD in tibial fracture models have been shown to be strongly associated with several indices of biomechanical and structural integrity indicative of the repair and healing processes [25,26].

## Conclusions

In conclusion, this trial provides Level-I evidence that use of LIPUS accelerates the healing process and likely improves the odds of achieving solid union in patients with delayed unions of the tibia. These positive findings should assist in establishing this non-invasive modality as a viable, effective treatment option for patients suffering these injuries.

## Competing interests

This study was supported, in part, by Smith & Nephew (Memphis, TN). JB is an independent consultant and received remuneration from the sponsor to assist in the development of the manuscript. The other authors declare that they have no competing interests.

## Authors' contributions

All authors read and approved the final manuscript. MS, JA and AS conceived of the study and participated in its design. JB had the primary role of interpreting the data analysis and authoring the manuscript. MS, JA and AS assisted in revising the manuscript for important intellectual content.

## Pre-publication history

The pre-publication history for this paper can be accessed here:

http://www.biomedcentral.com/1471-2474/11/229/prepub
